# Supplementary figures and images for: Carotenoid Accumulation in the Rhododendron chrysanthum Is Mediated by Abscisic Acid Production Driven by UV-B Stress
Source: Plants (Basel). 2024 Apr 9;13(8):1062. doi: 10.3390/plants13081062 (PMC11054193; doi:10.3390/plants13081062)

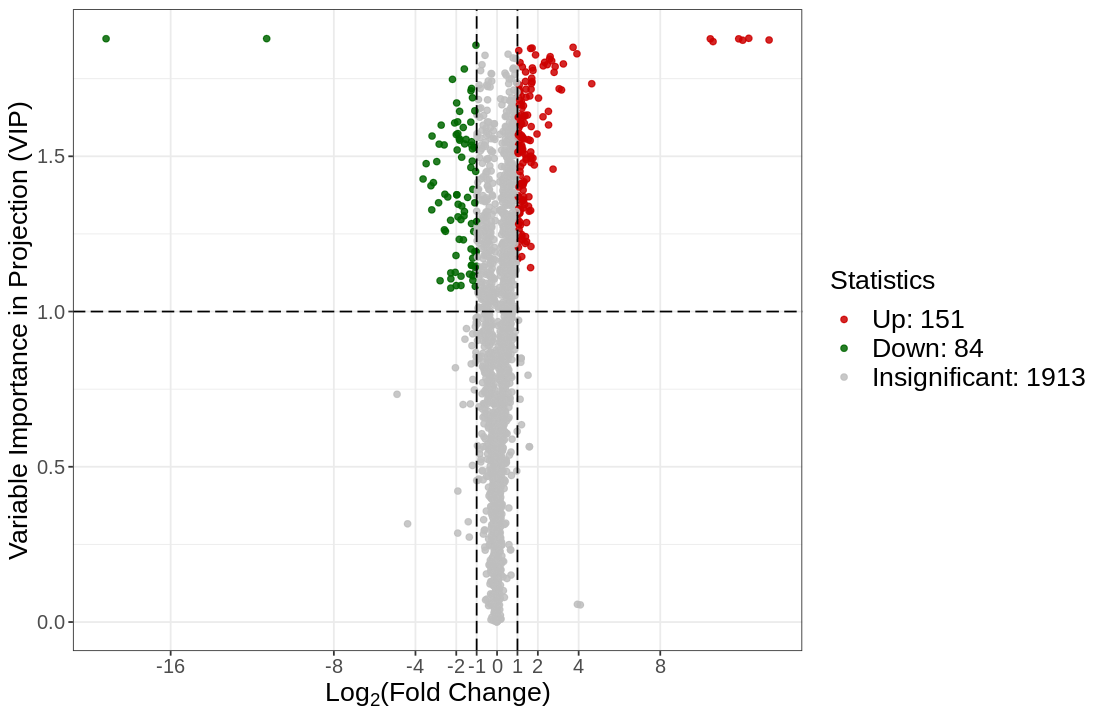

Supplement: Supplementary file 1 [file plants-13-01062-s001.zip › Supplementary materials/Figure S1.png]

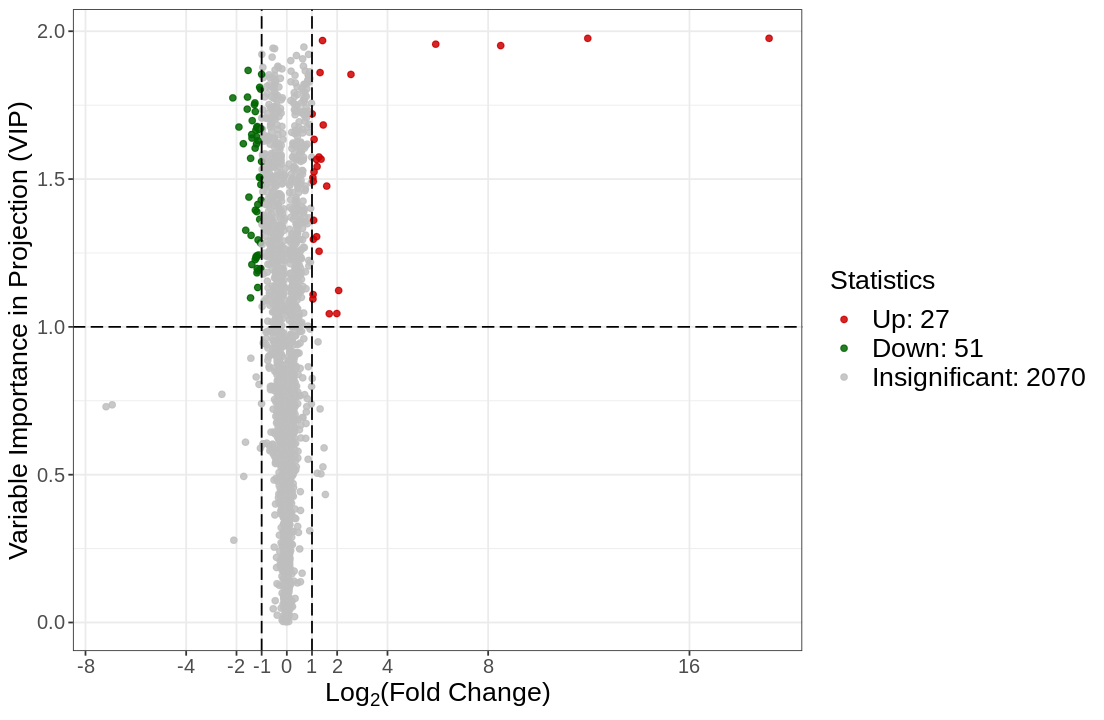

Supplement: Supplementary file 1 [file plants-13-01062-s001.zip › Supplementary materials/Figure S2 .png]
